# The complete chloroplast genome sequence of *Viola kunawarensis* Royle, a precious Uygur medicinal material

**DOI:** 10.1080/23802359.2022.2122882

**Published:** 2022-09-23

**Authors:** Jinchao Zhou, Jizhao Zhang, Yuanjin Qiu, Congzhao Fan

**Affiliations:** a State Key Laboratory of Soil Erosion and Dryland Farming on the Loess Plateau, Institute of Soil and Water Conservation, Northwest A&F University, Yangling, People’s Republic of China; b Xinjiang Institute of Chinese Materia Medica and Ethnical Materia, Xinjiang Key Laboratory of Chinese Materia Medica and Ethnic Materia Medica, Urumqi, People’s Republic of China

**Keywords:** Chloroplast genome, *Viola kunawarensis*, Illumina sequencing, phylogenetic analysis

## Abstract

*Viola kunawarensis* Royle is a precious Uygur medicinal material that has anti-fever and detoxifying effects. This study reports the complete chloroplast genome sequence of *V. kunawarensis* based on Illumina NovaSeq-PE150 platform sequencing reads. The genome is 156,837 bp long and contains a small single-copy (SSC) region of 17,059 bp and a large single-copy (LSC) region of 86,194 bp, separated by two inverted repeats (IRs) of 26,792 bp each. There are 111 unique genes in the chloroplast genome. In this study, *V. kunawarensis* was confirmed to be most closely related to all comprising *Viola* taxa except *Viola mirabilis* and *Viola websteri.*

*Viola kunawarensis* Royle 1839 is a perennial herb widely distributed in the Himalayas, the Pamir region, Tianshan, and the Tibet and Xinjiang autonomous regions of China (Chinese Academy of Science Flora of China Editorial Board 1991). The whole plant of *V. kunawarensis* is officinal, and it is included in the processing specification of traditional Chinese medicine in Xinjiang Uygur Autonomous Region. In Uygur traditional medicine, *V. kunawarensis* was once named *Viola tianshanica* Maxim., and it has been used to treat fever, cold, pleurisy, pneumonia, pharyngitis, sore, swelling, and carbuncles, among other conditions (Yu 2020). Modern research shows that *V. kunawarens*is has medicinal effects that include anti-inflammatory and antibacterial (Liang et al. 2015), cough relief, anti-phlegm, anti-asthma (Qu et al. 2011), and anti-tumor effects (Cao et al. 2014; Ji and Wei 2015), which have attracted much attention in recent years.

At present, *V. kunawarensi*s is the only source of medicinal materials which has been collected by “Processing specification of traditional Chinese medicine in Xinjiang Uygur Autonomous Region” in the genus *Viola*. Because of the limited availability and high price of the species, various species in *Viola* are used as substitutes for *V. kunawarensis* in the market, including *Viola odorata* and *Viola philippica* (Fan et al. 2019), which has seriously affected the safety and integrity of medicinal materials. However, owing to complex variation and interspecific heterogeneity, there are different opinions on the phylogeny of the genus *Viola* (Liang and Xing 2010; Sun et al. 2014). Therefore, it is extremely difficult to identify the basis of commercial *V. kunawarensis* medicinal materials by traditional morphological methods, which restricts the development of the industry. There were 11 species of published chloroplast genomes in the family Violaceae. In this study, the chloroplast genome of *V. kunawarensis* was assembled for the first time. The determination of this complete plastid genome sequence is a useful resource that will provide molecular data to illuminate its phylogenetic relationship within the genus and to better identify medicinal materials.

Plant materials of *V. kunawarensis* were collected from Taxkorgan County (75.4941°E, 37.2308°N), Xinjiang, China. The voucher specimen was deposited at the Xinjiang Institute of Chinese Materia Medica and Ethnical Materia (Urumqi, Congzhao Fan, 121653807@qq.com) with the voucher number 653131200614015LY. Genomic DNA was extracted using a DNeasy Plant Mini Kit (QIAGEN, Valencia, CA, USA) in this study. The Illumina NovaSeq-PE150 platform (Illumina, San Diego, CA, USA) was used to the genomic data of *V. kunawarensis*. The sequencing reads were assembled using MITObim v1.9 (Hahn et al. 2013), and the genome map was prepared using OGDraw online tools (Lohse et al. 2013).

The complete chloroplast genome was annotated using *Viola raddeana* (MH229818) as a reference. The chloroplast genome of *V. kunawarensis* has a circular quadripartite structure that resembles other angiosperm chloroplast genomes (Hahn et al. 2013). It has a length of 156,837 bp, containing a small single-copy (SSC) region of 17,059 bp and a large single-copy (LSC) region of 86,194 bp separated by two inverted repeats (IRs) of 26,792 bp each. The overall nucleotide composition is asymmetric (31.4% A, 32.4% T, 17.8% G, and 18.4% C) with AT content (63.8%) higher than GC content (36.2%).

The *V. kunawarensis* chloroplast genome contains 111 unique genes, including 77 protein-coding genes (PCGs), 4 rRNA genes, and 30 tRNA genes. In addition, 17 genes contain one or two introns, which includes 9 PCGs (*atp*F, *ndh*A, *ndh*B, *pet*B, *pet*D, *rpl*2, *rpl*16, *rpo*C1, *rps*12, and *ycf*3) possessing a single intron, 2 PCGs (*clp*P and *ycf*3) harboring two introns, and 6 tRNA genes (*trn*A-UGC, *trn*G-UCC, *trn*I-GAU, *trn*K-UUU, *trn*L-UAA, and *trn*V-UAC) harboring a single intron.

Geneious R11 was to. In total, 17 PCGs (atpA, *atp*B, *mat*K, *ndh*A, *ndh*D, *ndh*F, *ndh*H, *psa*A, *psa*B, *psb*A, *psb*B, *psb*C, *psb*D, *rbc*L, *rpo*A, *rpo*B, and *rpo*C1) which yielded reliable alignments were selected for a panel of 14 taxa (incl. 12 ingroup and 2 outgroup taxa). These PCGs were individually aligned and were then concatenated into a single alignment in Geneious R11. After that, the resultant alignment was exported into TOPALi v2.5 (Milne et al. 2009), and a phylogenetic tree was constructed with the program MrBayes v3.1.2 (Ronquist and Huelsenbeck 2003) as implemented in TOPALi v2.5. It was revealed that *V. kunawarensis* was relatively basal in the genus *Viola*. It was basal to the clade comprising all *Viola* taxa except *Viola mirabilis* and *Viola websteri,* Figure 1*.* These results would contribute to a better understanding of the genus *Viola* and provide molecular markers for the identification of *V. kunawarensis* and its congeners.

## Data Availability

We confirm that the complete mitogenome sequence can be accessed via NCBI GenBank accession no. OL606641 at https://www.ncbi.nlm.nih.gov. The associated BioProject, SRA, and Bio-Sample numbers are PRJNA791966, SRR17326421, and SAMN24371149, respectively. Phylogeny of the genus *Viola* based on the Bayesian phylogenetic analysis of the plastid protein-coding genes. The support values next to the nodes are Bayesian posterior probabilities according to the Bayesian analysis. The outgroup taxa used in this study are two species within the family Passifloraceae, i.e. *Adenia mannii* and *Passiflora nitida*.
